# Orchestrating liver development

**DOI:** 10.1242/dev.114215

**Published:** 2015-06-15

**Authors:** Miriam Gordillo, Todd Evans, Valerie Gouon-Evans

**Affiliations:** 1 Department of Surgery, Weill Cornell Medical College, New York, NY 10065, USA; 2 Department of Developmental and Regenerative Biology, Black Family Stem Cell Institute, Icahn School of Medicine at Mount Sinai, New York, NY 10029, USA

**Keywords:** Cholangiocyte, Hepatoblast, Hepatocyte, Liver development, Transcription factors

## Abstract

The liver is a central regulator of metabolism, and liver failure thus constitutes a major health burden. Understanding how this complex organ develops during embryogenesis will yield insights into how liver regeneration can be promoted and how functional liver replacement tissue can be engineered. Recent studies of animal models have identified key signaling pathways and complex tissue interactions that progressively generate liver progenitor cells, differentiated lineages and functional tissues. In addition, progress in understanding how these cells interact, and how transcriptional and signaling programs precisely coordinate liver development, has begun to elucidate the molecular mechanisms underlying this complexity. Here, we review the lineage relationships, signaling pathways and transcriptional programs that orchestrate hepatogenesis.

## Abstract (precis)

**Summary:** This review summarises the complex interplay between cellular lineages, signalling pathways, and transcriptional programs necessary to form a vertebrate liver.

## Introduction

**Fig. 1.**
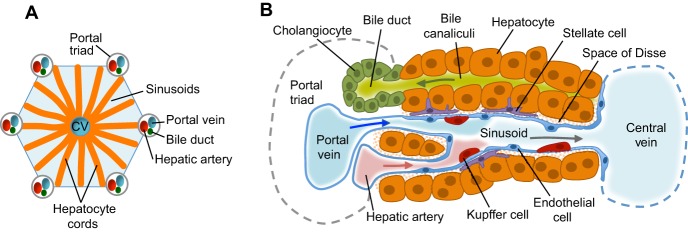
**Liver structure and cell types.** (A) The liver is organized from many lobules, which constitute its functional units. Each lobule is composed of a central vein (CV), from which hepatocyte cords radiate towards portal triads. The portal triad consists of a portal vein, hepatic artery and biliary duct. Hepatocyte cords are single-cell sheets of hepatocytes separated by sinusoids that carry blood from the portal triads to the central vein. (B) Within each lobule are a number of sinusoids, which are discontinuous vessels built from specialized fenestrated endothelial cells of the liver. Stellate (or Ito) cells are located in the space of Disse between the hepatocyte cords and sinusoids. Kupffer cells, which are the specialized macrophages of the liver, also reside in sinusoids. Hepatocytes secrete bile salts into the bile canaliculi that lead to the bile duct. Cholangiocytes are the epithelial cells lining the bile ducts.

The liver controls glycolytic and urea metabolism, blood detoxification and cholesterol levels, while supporting the hematopoietic and digestive systems. Given that it is the largest and most toxin-exposed organ in the body, it is not surprising that liver diseases, including fibrosis, cirrhosis, hepatitis and hepatocarcinoma, are major contributors to morbidity and mortality. Liver histology belies a complex and highly organized architecture (Fig. 1) incorporating numerous cell types and tissue interactions that are orchestrated from the earliest stages of embryonic development. Indeed, studies of animal models have identified key signaling pathways and tissue interactions that progressively generate the progenitors, lineages and functional tissues of the liver (Si-Tayeb et al., 2010; Zorn and Wells, 2007). As such, many of the cellular constituents, signaling pathways and transcriptional regulators required to build a functional liver during embryogenesis are now known. Recent progress using conditional genetics, novel imaging strategies and genome-wide bioinformatics has furthered our understanding of how these various signaling, transcriptional and epigenetic programs coordinate liver development. Embryonic stem cell (ESC) models have also helped, particularly in understanding early human liver development. It is now possible to model liver development and disease ‘in a dish’, and this offers a model for understanding how functional organogenesis is accomplished during normal development.

**Fig. 2.**
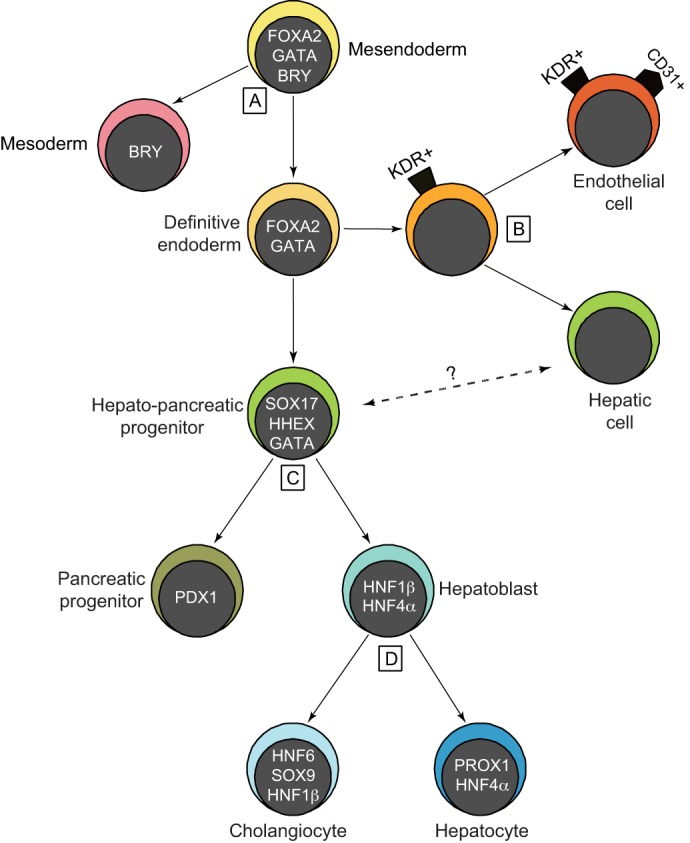
**Bipotential progenitors progressively generate hepatic lineages.** Four key transition points (A-D) during fetal liver development are highlighted. (A) Bipotential mesendoderm cells segregate into brachyury (BRY)^+^ mesoderm and definitive endoderm (DE). The prospective foregut endoderm maintains expression of the pioneer transcription factors FOXA2 and GATA4/6 (denoted GATA). (B) Studies of ESC cultures suggest that the DE includes a subset of KDR^+^ progenitors that generate and support the development of hepatic cells; they can also give rise to CD31 (PECAM1)^+^ endothelial cells. (C) Foregut DE generates a bipotential hepato-pancreatic progenitor (SOX17^+^, HHEX^+^, GATA^+^) that produces both PDX1^+^ pancreatic progenitors and HNF1β^+^, HNF4α^+^, PDX1^–^ hepatoblasts. It is not known how closely the hepatic cells derived from KDR^+^ endoderm are related to this cell (question mark). (D) Finally, the hepatoblast is a bipotential progenitor for both cholangiocytes (bile duct cells; HNF6^+^, SOX9^+^, HNF1β^+^) and hepatocytes (PROX1^+^, HNF4α^+^). Note that the figure only indicates when factors initially function for transitions; in many cases (such as for FoxA and GATA factors) they continue to be expressed and also function at later stages.

A common theme that has emerged from these multiple experimental approaches is the progressive commitment of bipotential progenitors to one or other fate during liver development (Fig. 2), often using the same signaling pathways but at different developmental stages or in different cellular contexts. This simplified concept might help clarify the mechanistic details of how cell lineages and tissue architecture in general are coordinated. Building on this theme, we first provide a general overview of the stages involved in liver development. We then review the signaling pathways, transcription factors and epigenetic regulators that govern cell fate choices during liver development, from the initial specification of endoderm to the maturation of functional hepatocytes.

## An overview of liver lineages, development and morphogenesis

The mature liver is composed of lobes (four in human) containing multiple cell types, including hepatocytes, cholangiocytes, endothelial cells, stellate cells and Kupffer cells. The functional histologic unit of the liver is the liver lobule, which consists of polygonal cords of liver cells arranged around a central vein that is a terminal branch of a hepatic vein. Branches of the portal vein, hepatic artery and biliary tree are located at the periphery of the central vein (Fig. 1). Hepatocytes are the main parenchymal cell of the liver, constituting ∼80% of the mass. They are cuboidal and secrete serum albumin, bile and crucial blood clotting factors. The epithelial cells that line the bile ducts are termed cholangiocytes. Here, we refer mostly to intrahepatic bile ducts, although it should be noted that cholangiocytes function throughout the biliary tree. Below, we provide an overview of hepatic specification, liver bud development and the formation of hepatoblasts, which act as a common progenitor for hepatocytes and cholangiocytes. Further details concerning stellate cells and Kupffer cells can be found in Boxes 1 and 2, respectively.

Box 1. **Stellate cells**

Stellate cells located in the space of Disse constitute the major mesenchymal component of the liver. When quiescent, they are the main reservoir of vitamin A. When activated by injury or infection, they differentiate into αSMA-expressing myofibroblast-like cells capable of depositing ECM and directing temporary fibrotic scar formation (Puche et al., 2013). One source of stellate cells during development is the septum transversum-derived mesothelium (reviewed by Yin et al., 2013). However, stellate cells express markers from all three germ layers and are closely associated with sinusoidal endothelium, so they might be derived from additional sources. In zebrafish, stellate cells are marked by a *hand2:EGFP* transgenic reporter, consistent with either a mesodermal or neural crest origin (Yin et al., 2012). They associate with the liver surface at ∼64 hpf, entering after sinusoidal endothelial cells invade, and the two cell types closely associate. However, their development and migration are independent of vascular cells, as shown in *cloche* mutants. Subsequently, stellate cells regulate hepatic vascular development, functioning as a pericyte in the quiescent state. Both *Wt1* and *Lhx2* are expressed in quiescent stellate cells. *Wt1* and *Lhx2* knockout embryos develop stellate cells but exhibit an ectopic activation state with ECM deposition, fibrosis and a smaller liver due to hepatoblast proliferation defects, perhaps owing to aberrant levels of cytokines, including HGF and FGF10. Quiescence in stellate cells is also dependent on the Wnt pathway; mesenchymal knockout of β-catenin generates a liver phenotype with activated αSMA-expressing stellate cells and dilated sinusoids (Berg et al., 2010; Kordes et al., 2008). Stellate cells also express *Jag1*, which is essential for biliary cell development and can stimulate the differentiation and maturation of hepatocytes *in vitro* (Nagai et al., 2002). Finally, stellate cells express SDF1 (CXCL12), a cytokine that is required for vascular integrity and for recruiting CXCR4-expressing hematopoietic progenitors into the fetal liver.

Box 2. **Kupffer cells**

Kupffer cells (KCs) are liver macrophages that reside in the sinusoids, constituting 15% of liver cells (Naito et al., 2004). KCs originate from fetal yolk sac precursors and self-renew dependent on GM-CSF and M-CSF (Schulz et al., 2012; Yona et al., 2013). Although there is no direct evidence for KCs regulating hepatogenesis, they are likely to impact fetal liver erythropoiesis. During chronic injury, Wnt ligands released by KCs during phagocytosis can direct bipotential progenitors to the hepatocyte fate (Boulter et al., 2012). Furthermore, KCs play an important role in liver regeneration by influencing the invasive behavior of liver progenitor cells (Van Hul et al., 2011).

### Endoderm specification

The two main liver cell types, namely hepatocytes and cholangiocytes, are derived from endoderm that emerges from the anterior primitive streak of the gastrulating embryo and is identifiable by the shield stage 6 h post fertilization (hpf) in zebrafish, at embryonic day (E) 7.5 in mouse, and in the third week of human gestation (Table 1). Studies of ESC models indicate that, as these cells migrate toward the anterior of the embryo, they segregate from bipotential mesendoderm to form definitive endoderm (DE), a monolayer of cells on the ventral aspect of the developing embryo. The DE then forms a tube as the murine embryo rotates along the anterior-posterior (AP) axis and is patterned into three progenitor domains that, in mammals, comprise the foregut, midgut and hindgut. The foregut endoderm then gives rise to the liver along with the ventral pancreas, stomach, lungs and thyroid (Tremblay and Zaret, 2005).

**Table 1.**
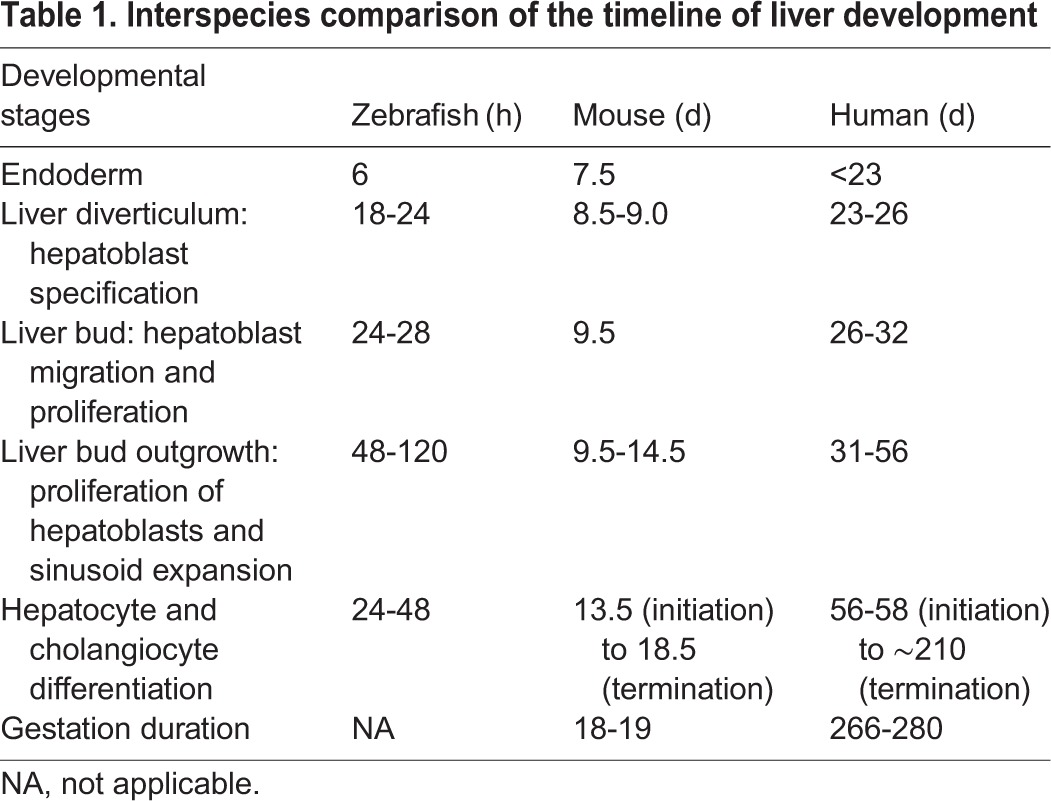
**Interspecies comparison of the timeline of liver development**

### Liver diverticulum formation and budding

**Fig. 3.**
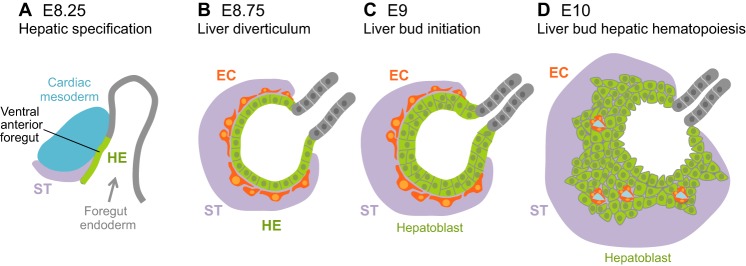
**Liver diverticulum and bud formation in mouse.** (A) Sagittal section of the cephalic portion of the E8.25 mouse prospective hepatic endoderm (HE, green). The convergence of the cardiac mesoderm (blue) and septum transversum (ST, purple) is required for hepatic specification. (B-D) Transverse sections of the mouse liver diverticulum progressing to the liver bud stage. (B) At E8.75, endothelial cells (ECs, orange) are found surrounding the thickened hepatic endoderm, which initiates a budding process into the septum transversum. Endothelial cells contribute to hepatic specification. (C) At E9, the hepatic endoderm transitions from a columnar to a pseudostratified epithelium. (D) At E10, hepatic endoderm cells, identified as hepatoblasts, proliferate and migrate into the septum transversum to form the liver bud. Endothelial cells are also required for liver bud formation. Hematopoietic progenitor cells now start to migrate into the bud to establish liver fetal hematopoiesis.

The process of liver diverticulum (liver bud) organogenesis is remarkably well conserved across vertebrate species. By E9.0 in mice, the ventral domain of the foregut adjacent to the cardiac mesoderm and septum transversum thickens to form the liver diverticulum (Fig. 3A,B). Subsequently (Fig. 3C,D), the diverticulum thickens and transitions from a monolayer of cuboidal endoderm cells into a multilayer of pseudostratified cells called hepatoblasts, which delaminate, proliferate and invade the surrounding septum transversum to form the liver bud (Bort et al., 2006). Hepatoblasts are marked by the expression of alpha-fetoprotein (AFP) and albumin (ALB) transcripts and proteins (Cascio and Zaret, 1991; Nava et al., 2005; Schmid and Schulz, 1990; Shiojiri, 1981).

In zebrafish, by 24 hpf endoderm cells form a solid bar of midline cells called the intestinal rod (Field et al., 2003). Liver morphogenesis is then initiated by a thickened epithelial structure that buds away from the ventral side of the rod, around the position of the first somite, rather than by migratory invasion. By 28 hpf, two endoderm-thickened domains are apparent; the more posterior domain contributes to the pancreas, and the anterior domain to the liver. In humans the first liver cells are visible in the liver plate, which is located close to the caudal heart, from day 23 of gestation (Hutchins and Moore, 1988; Severn, 1971).

### The role of endothelial cells during liver morphogenesis

Explant studies have shown that hepatic specification requires the surrounding cardiac mesoderm (Gualdi et al., 1996; Le Douarin, 1975), septum transversum (Rossi et al., 2001) and (in mammals) endothelium. Endothelial cells form a necklace around and adjacent to the mouse liver diverticulum at E9.0, and the ablation of endothelial cells (as occurs in VEGF receptor-deficient mouse embryos) blocks liver budding (Matsumoto et al., 2001). Endothelial cells are also important for hepatic specification from murine endoderm cells, and for expanding hepatoblasts from ESC-derived or embryonic endoderm cells (Han et al., 2011). Some endothelial cells derive from a subset of bipotential KDR (FLK1, VEGFR2)^+^ endoderm cells (Goldman et al., 2014). It has been noted that a bud forms in the zebrafish *cloche* mutant, which lacks endothelial cells, suggesting that the initial steps of liver bud formation in zebrafish are not dependent on vascularity and probably do not involve this bipotential hepato-vascular progenitor (Field et al., 2003). Regardless, the zebrafish liver also depends on a vascular system that invades the hepatic tissue starting at ∼66 hpf and is crucial for patterning of the hepatic architecture including bile ducts and hepatocyte polarization (Sakaguchi et al., 2008).

## From hepatoblast to hepatocyte

Following their specification and migration into the septum transversum, hepatoblasts undergo proliferation and differentiate into hepatocytes and cholangiocytes. This process has been studied in mouse and zebrafish embryos and *in vitro* using populations of isolated hepatoblasts. Together, these studies have revealed a number of factors that mark hepatoblasts and have provided insights into how this cell population expands and transitions into functional hepatocytes.

### Hepatoblast markers and purification

Bipotential hepatoblasts express AFP as well as markers for both hepatocytes [ALB, HNF4α, keratin 18 (CK18 or KRT18)] and cholangiocytes (CK19). During the isolation of hepatoblasts from fetal mouse liver, hematopoietic cells that are positive for CD45 (PTPRC; leukocytes) or TER119 (LY76; erythrocytes) are excluded. The CD45^−^, TER119^−^, cKIT^−^, CD29 (ITGβ1)^+^, CD49f (ITGα6)^+^ cell population contains a hepatic colony-forming unit (H-CFU-C) that is able to differentiate into hepatocytes and cholangiocytes (Suzuki et al., 2000), and related alternative sorting strategies have been described (Kakinuma et al., 2009; Kamiya et al., 2009; Minguet et al., 2003; Suzuki et al., 2003). In parallel, specific markers for hepatoblasts include Dlk1 (Pref1) (Tanimizu et al., 2003), E-cadherin (cadherin 1) (Nierhoff et al., 2005; Nitou et al., 2002), Liv2 (Watanabe et al., 2002), CD24a, Nope (Igdcc4) (Nierhoff et al., 2007) and EpCAM (Tanaka et al., 2009). Purified hepatoblasts can differentiate into hepatocytes *in vivo* following transplantation into rodent livers (Miyajima et al., 2014). Hepatoblasts and hepatic progenitor cells have also been found in developing human fetal livers during the first two trimesters of gestation, and these have been shown to express various factors (Table 2) including ALB, AFP, CK14, CK18, CK19, DLK1, E-cadherin, EpCAM, CD133 (PROM1) and the hepatocyte-specific antigen HepPar1 (Haruna et al., 1996; Terrace et al., 2007; Wauthier et al., 2008).

**Table 2.**
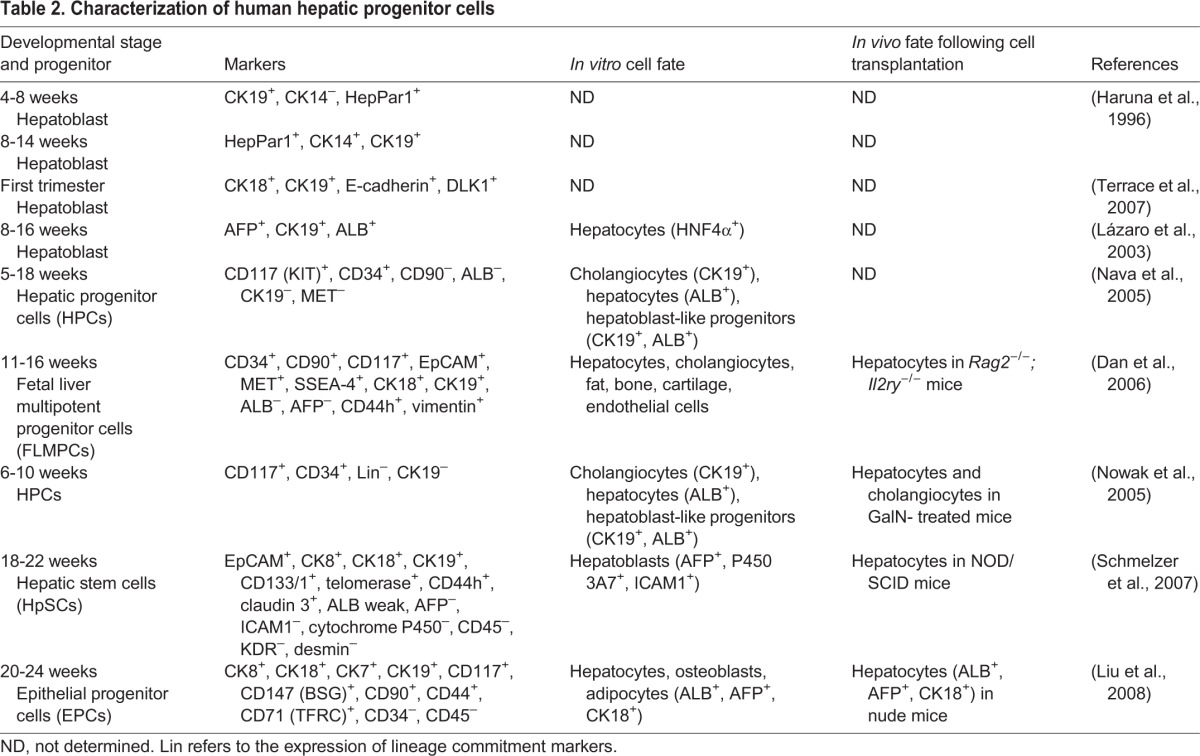
**Characterization of human hepatic progenitor cells**

### Fetal hepatic stem cells

Within the pool of human hepatoblasts is a subset of cells that exhibit a stem cell-like signature (Wauthier et al., 2008). These hepatic stem cells are located along the fetal liver ductal plate and, during *in vitro* culture, exhibit a self-renewal capacity of over 150 population doublings and the capacity to generate hepatoblasts (Schmelzer et al., 2007). Unlike hepatoblasts, they are negative/low for AFP and ALB; instead, they express stem cell markers including CD133, CD34 and cKIT (Nava et al., 2005). They share some hepatoblast markers such as EpCAM, but are distinguished by the expression of NCAM1 and claudin 3. A mesenchymal stem cell phenotype is suggested by their expression of CD90 (THY1) and vimentin and by their ability to differentiate *in vitro* into fat, bone, cartilage and endothelial cells. The ability of these stem cells to differentiate to hepatocytes *in vivo* has been shown using *Rag2*^−/−^*;Il2ry*^−/−^ (Dan et al., 2006), GalN-treated (Nowak et al., 2005), NOD/SCID (Schmelzer et al., 2007) or nude mice (Liu et al., 2008), although lineage-tracing studies *in vivo* did not find evidence that ductal plate cells transition through a hepatoblast progenitor (Carpentier et al., 2011).

Similar cells have also been identified in the mouse fetal liver that express DLK1, continuously self-renew, and differentiate *in vitro* into both hepatocytes and cholangiocytes (Tanimizu et al., 2003). Hepatic stem cell lines derived from dissociated fetal livers can persist long-term *in vitro* and contribute to hepatocyte and cholangiocyte lineages following transplantation into albumin-urokinase plasminogen activator/severe combined immunodeficiency (Strick-Marchand et al., 2004) or carbon tetrachloride-injured (Tsuchiya et al., 2005) mouse models. Although their ontogeny is unclear, a heterogeneous population of bipotential progenitors was originally identified in adult rodent livers (Farber, 1956) that, owing to the shape of their nuclei, are referred to as ‘oval’ cells. The origin and function of such putative ‘stem cell’ populations in the liver has been a topic of considerable controversy and is discussed further in Box 3.

Box 3. **Liver stem/progenitor cells**

A number of other cell types have long been considered candidates for facultative stem or progenitor cells for liver homeostasis and regeneration, when hepatocyte proliferation is compromised. Ductal plate cells, for example, are Sox9^+^ embryonic biliary precursors. They are remodeled into intrahepatic bile ducts and can generate periportal hepatocytes and oval cells (Carpentier et al., 2011). Hepatoblasts are specified embryonic liver cells that are bipotential for hepatocytes and cholangiocytes. A subset of hepatoblasts that express markers found in stem cells, such as CD133 and cKIT, have been referred to as hepatic stem cells. They are proliferative, bipotential and can generate hepatocytes when transplanted. In contrast to these embryonic/fetal progenitors, oval cells arise from the biliary tree in the adult liver following damage. This regeneration following injury is often referred to as a ‘ductal reaction’. The oval cells are identified by the cell surface markers MIC1-1C3 (Dorrell et al., 2008), EpCAM or TROP2 (TACSTD2) (Okabe et al., 2009), CD133 (Kamiya et al., 2009; Rountree et al., 2007; Suzuki et al., 2008), CD24 (Qiu et al., 2011) and by the transcription factors SOX9 (Dorrell et al., 2011; Furuyama et al., 2011), FOXL1 (Shin et al., 2011) and LGR5 (Huch et al., 2013). A few studies have shown some contribution of oval cells to the hepatocyte lineage following transplantation in liver-deficient mouse models. However, recent murine lineage-tracing studies failed to find any significant contribution of hepatocytes derived from any cells other than hepatocytes themselves, following commonly used injury-induced regeneration (Rodrigo-Torres et al., 2014; Schaub et al., 2014; Tarlow et al., 2014; Yanger et al., 2014). The establishment of novel animal models with liver deficiency that closely recapitulate human liver diseases might be needed to reveal evidence of functional adult liver stem cell populations, if indeed they do exist (Grompe, 2014).

### Hepatoblast proliferation and expansion

The fetal liver is a major transitional site for mammalian hematopoiesis (Golub and Cumano, 2013). Consequently, hepatoblasts and hematopoietic progenitor cells develop together from the time the murine liver bud is formed, at E10 in mice or during the fifth week of gestation in humans (Migliaccio et al., 1986). The expansion and proliferation of hepatoblasts is influenced by these and other transient cells. For example, the interleukin-6 family member oncostatin M (OSM), which is secreted by mouse hematopoietic progenitors, can induce *in vitro* hepatoblast proliferation (Kamiya et al., 1999) and regulate homophilic cell adhesion by inducing E-cadherin-based adherent junctions (Matsui et al., 2002). Since OSM is dispensable for normal liver development there might be redundancy for receptor signaling, for example by IL6 (Nakamura et al., 2004). The zebrafish liver is not a hematopoietic organ, so the developmental signals that are provided by mammalian fetal-stage blood cells must come from other sources in zebrafish. As the liver bud expands, a monolayer of immature mesothelial cells expressing high levels of podocalyxin-like protein 1 (PCLP1, or PODXL) appears at E10.5 lining the developing murine liver lobes. Co-culture experiments have shown that these promote the expansion of hepatoblasts in a paracrine manner, until acquiring a more mature phenotype with mesothelin expression and loss of PCLP1 (Onitsuka et al., 2010). Finally, using human ESC (hESC) differentiation cultures, it was shown that KDR-expressing progenitors for hepatoblast-like cells (hepatic cells) arise concomitantly with committed hepatic cells and promote their specification (as indicated by AFP expression) and maturation (as indicated by ALB expression), and this function can be blocked by inhibiting KDR activity (Goldman et al., 2013). Furthermore, the KDR^+^ progenitors can generate both hepatic cells and functional endothelium (Goldman et al., 2014).

### Hepatoblast differentiation: the generation of hepatocytes and cholangiocytes

**Fig. 4.**
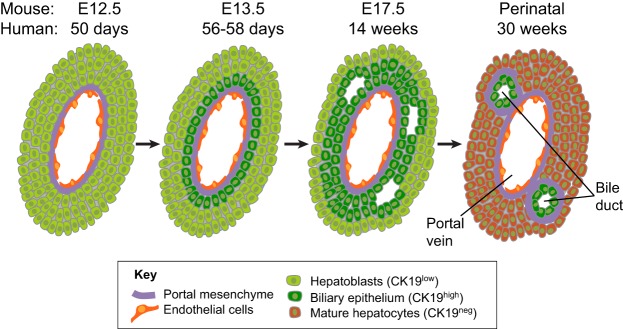
**Bile duct development.** Prior to bile duct development, hepatoblasts are the only epithelial progenitor cells in the fetal liver. Ductal plate formation is the first sign of bile duct development. It is initiated at E13.5 in mice and at around day 56-58 after fertilization in humans. The ductal plate is composed of a monolayer of cholangiocyte precursors derived from hepatoblasts in contact with the portal mesenchyme. These precursors express high levels of CK19 compared with hepatoblasts that are located further away from the portal veins. At ∼E17.5 in mice, and during the second trimester in humans, the ductal plate duplicates. Luminal pockets develop between the two layers of precursors. In the perinatal period in mice and at ∼30 weeks of gestation in humans, some of these pockets form bile ducts composed of mature cholangiocytes that maintain CK19 expression. The remaining ductal plate areas regress in a process called ductal plate remodeling. The hepatoblasts located away from the portal veins differentiate into mature hepatocytes that completely downregulate CK19.

Hepatoblasts start differentiating into hepatocytes and cholangiocytes at ∼E13.5 in mouse, 24 hpf in zebrafish and 56-58 days of gestation in humans (Table 1). In humans, cholangiocyte precursors are generated at the ductal plate from a monolayer of hepatoblasts surrounding the portal veins (Ruebner et al., 1990), whereas hepatoblasts located away from portal vein areas differentiate into hepatocytes (Fig. 4A-D). The cholangiocyte precursors express higher levels of CK19 than adjacent hepatoblasts, become cuboidal or columnar and form a bilayer of CK19^+^ cells (Fig. 4B,C). Subsequently, CK19 expression becomes restricted to cholangiocytes, which eventually bud off from the ductal plate to form tubules at ∼14 weeks of gestation in humans (Haruna et al., 1996) and E17.5 in mice (Fig. 4C). During this remodeling process, the remaining portion of the ductal plate regresses. Cholangiocyte differentiation then terminates at ∼30 weeks of gestation in humans and during the neonatal period in rodents (Terada and Nakanuma, 1993) (Fig. 4D), although some ductal plate cells may persist in neonates, children and even adults. The subsequent development of the biliary tract has been thoroughly reviewed elsewhere (Lemaigre, 2010).

### Hepatocyte maturation and heterogeneity

Hepatocytes constitute a heterogeneous population, and this heterogeneity is thought to arise via a phenomenon termed ‘metabolic zonation’ (Jungermann and Kietzmann, 1996, 2000), whereby liver functions are compartmentalized in the hepatic lobule. The first row of periportal hepatocytes, surrounding the portal space, receives a mixture of blood from both the hepatic artery and the portal vein and is in contact with the bile ducts. On the opposite side of the liver cell plate, the hepatocytes surround a hepatic centrilobular vein and are called perivenous hepatocytes. This organization gives rise to classic zonal functions of the liver and is reflected in the compartmentalized localization of key enzymes and transporters in the lobule. The expression of these enzymes and transporters, and hence the function of hepatocytes, changes throughout development (Moscovitz and Aleksunes, 2013). In addition, the prenatal liver plasma membrane exhibits decreased phosphatidyl choline:phosphatidyl ethanolamine ratios and increased sphingomyelin:phosphatidyl choline ratios. During the last weeks of gestation, the inhibition of glycolytic enzymes, coupled with the rise in gluconeogenic enzyme levels, reflects maturation of the liver from a primarily glycolytic role in the first two trimesters to a gluconeogenic role shortly before birth. Hepatocyte maturation continues after birth, with an age-dependent reduction in lipid:protein ratio and increasing membrane cholesterol that decreases membrane fluidity (Devi et al., 1992).

## The regulation of liver development by cell signaling pathways

**Fig. 5.**
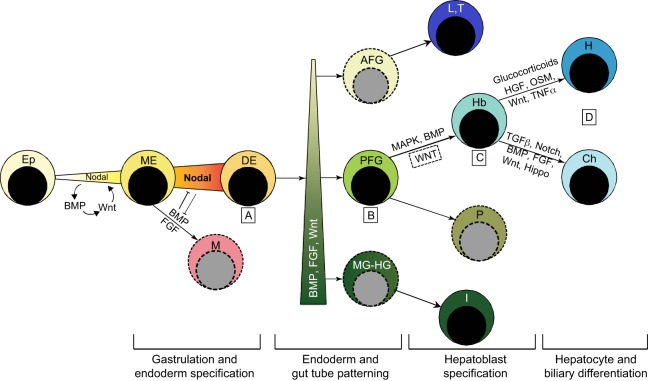
**Key signals mediating progenitor fate decisions during hepatogenesis.** (A) Activation of Nodal signaling in the embryonic epiblast (Ep) lineage initiates mesendoderm (ME) formation. In *Xenopus* and zebrafish, maternal signals activate this pathway. In mice, Nodal, BMP4 and WNT3 act in a reinforcing loop to activate Nodal and induce ME. Subsequently, high levels of Nodal signaling establish the endoderm regulatory network leading to the segregation of, and commitment to, definitive endoderm (DE), while FGF and BMP drive mesoderm (M) formation by antagonizing Nodal signaling. (B) The graded activity of Wnt, FGF and BMP signaling patterns the endoderm along the AP axis to generate posterior foregut precursors (PFG) with hepato-pancreatic potential that can be distinguished from anterior foregut [AFG; deriving lung (L) and thyroid (T)] and midgut-hindgut [MG-HG; deriving intestine (I)] progenitors. P represents pancreatic progenitors. (C) FGF-MAPK, BMP and Wnt signaling positively regulate hepatic specification to generate hepatoblasts (Hb), although the role of Wnt at this stage of liver development has not been demonstrated in mammals (indicated by dashed box). (D) Hepatoblast differentiation into cholangiocytes (Ch) and hepatocytes (H), and the final maturation of these cells, is regulated by a wide array of signaling pathways that display complex cross-regulation. These pathways also influence the 3D structural organization of the liver and define its zonal characteristics. The corresponding developmental stages are indicated beneath the scheme.

Morphogenetic movements expose cells to different signaling centers during liver development. The key signaling pathways used during hepatogenesis include those controlled by transforming growth factor β (TGFβ), Wnt, fibroblast growth factor (FGF), Notch and bone morphogenetic protein (BMP) ligands (Fig. 5), some of which give rise to opposing cellular responses at consecutive developmental stages (Zaret and Grompe, 2008). In recent years, the mechanisms by which these signaling pathways influence the various stages of liver development have been elucidated. These findings have guided the development of protocols to derive endoderm and hepatocyte populations *in vitro* by replicating the highly dynamic developmental signaling process and by progressively directing lineage bifurcations (Gouon-Evans et al., 2006; Loh et al., 2014).

### Signaling during endoderm specification

At the center of DE specification is the Nodal signaling pathway. Nodals are members of the TGFβ family of growth factors that signal via type I [Alk4 (Acvr1b) or Alk7 (Acvr1c)] and type II [ActRIIA (Acvr2a) or ActRIIB (Acvr2b)] activin receptors and the co-receptor Cripto (Tdgf1) to drive mesendoderm formation, followed by the segregation and commitment of the endoderm and mesoderm layers (Shen, 2007). In all vertebrates, this pathway is necessary and sufficient for endoderm formation (Fan et al., 2007; Hyde and Old, 2000; Schier and Talbot, 2005; Xanthos et al., 2001). In zebrafish, a signal from the extra-embryonic yolk syncytial layer activates Nodal signaling to specify mesendoderm progenitors. In mice, uncleaved Nodal precursor in the epiblast activates the expression of BMP4 and the Nodal proprotein convertases furin and SPC4 (PCSK6) in the extra-embryonic ectoderm (ExE). These proconvertases accelerate local mature Nodal expression through an autoregulatory loop, while BMP4 in the ExE activates *Wnt3* expression in the posterior epiblast and visceral endoderm. In turn, Wnt signaling maintains high levels of Nodal selectively in the posterior proximal epiblast, thereby initiating primitive streak formation and mesendoderm induction (Ben-Haim et al., 2006).

Whereas high levels of Nodal promote endoderm, low levels promote mesoderm (Grapin-Botton and Constam, 2007; Vincent et al., 2003), and an autoregulatory loop maintains the expression, processing and secretion of Nodals and Nodal antagonists to reinforce and sustain endoderm specification. Deletion of β-catenin in the anterior primitive streak leads to ectopic cardiac mesoderm at the expense of endoderm, indicating that Wnt signaling helps segregate mesoderm and endoderm from mesendoderm precursors (Lickert et al., 2002). Segregation is also mediated by mutual antagonism of the endoderm and mesoderm gene regulatory networks. FGF and BMP signaling antagonize Nodal signaling, for example by controlling the transcriptional activity of Sox32 (Cas), a key endoderm transcription factor; FGF/ERK signaling attenuates Sox32 transcriptional activity via phosphorylation, while BMP induces the expression of Vox, which binds and represses Sox32 (Mizoguchi et al., 2006; Poulain et al., 2006; Zhao et al., 2013). Mutual antagonism between BMP and Nodal signaling also regulates the development of foregut DE, both in mice (Yang et al., 2010) and during endoderm specification from ESC-derived mesendoderm (Loh et al., 2014), although the molecular mechanism underpinning this antagonism is unknown.

### Signaling during endoderm patterning

Following gastrulation, the endoderm is broadly patterned along the AP axis (Horb and Slack, 2001; Kimura et al., 2007), becoming progressively subdivided into territories associated with specific organ lineages by the combined and graded activity of Wnt, FGF and BMP signaling, which represses foregut identity and promotes hindgut fate (Zorn and Wells, 2009). During the gastrula and early somite stages, mesoderm-derived Wnts, including Wnt5a/5b/8/11, induce high nuclear β-catenin activity that promotes the expression of posterior endoderm genes while repressing anterior genes (Goessling et al., 2008; McLin et al., 2007; Sherwood et al., 2011). By contrast, low levels of β-catenin activity at the anterior side, regulated by the expression of secreted Wnt antagonists such as secreted frizzled-related protein 1 (SFRP1), SFRP2, SFRP3 (FRZB), SFRP5, Crescent and DKK1 are necessary and sufficient to maintain foregut identity. For example, SFRP5 expression is essential for foregut formation in *Xenopus* and zebrafish, acting via local inhibition of canonical and non-canonical Wnt11 signaling (Li et al., 2008; Stuckenholz et al., 2013). In *Xenopus*, the Wnt receptor FZD7 stimulates low levels of canonical and non-canonical Wnt activity essential for coordinating foregut progenitor proliferation and gene expression (Zhang et al., 2013). SFRP5, Wnt ligand and FZD7 interactions thus regulate differential thresholds of Wnt/β-catenin and Wnt/JNK signaling that coordinate endoderm progenitor fate, proliferation and morphogenesis.

BMP-induced patterning of the endoderm is also dynamic and involves multiple ligands expressed in the adjacent mesenchyme, receptors expressed in the endoderm and extracellular modulators including the BMP antagonists chordin and noggin. BMP activity is required for Wnt and FGF signaling to initiate posterior gene expression, and it must be blocked in the DE for anterior specification (Green et al., 2011). As with Wnt signaling, it is not the absence or presence of BMP signaling but an appropriate level of activity that is essential for foregut development. FGF signaling, by contrast, maintains cells in a state of responsiveness to Wnt and BMP signals.

### Signaling pathways controlling hepatic specification

Following the formation of the foregut endoderm, the onset of hepatogenesis requires FGF, BMP and Wnt from the surrounding mesoderm to induce hepatic specification. Accordingly, murine endoderm explants initiate hepatic (rather than pancreatic) gene expression when cardiac tissue is included, or when FGF1 or FGF2, but not FGF8, is added to the culture (Deutsch et al., 2001; Jung et al., 1999). However, FGF1 and FGF2 are not required for liver development, and only FGF8 and FGF10 are expressed in cardiac mesoderm. Prehepatic and hepatic endoderm cells express FGFR1, FGFR2 and FGFR4, and the MAPK pathway is the downstream signaling branch implicated in hepatic specification (Calmont et al., 2006; Shin et al., 2007). In zebrafish, FGF signaling is essential for liver specification (Shin et al., 2007). Furthermore, in whole mouse embryo cultures, a reduction in FGF signaling impairs gene expression and morphogenesis of the anterior portion of the liver, with little impact on the posterior bud, suggesting that FGF signaling is differentially required along the AP axis (Wang et al., 2015). The anterior bud is closer to the putative source of FGF (the heart), although no differences in the expression of pathway components or in the distribution of FGF-MAPK signaling activity along the AP axis are known (Calmont et al., 2006; Wang et al., 2015). The anterior and posterior liver precursors might thus acquire an AP pattern before specification and therefore respond differentially to inductive signals. In support of this, it was shown that the liver bud derives from both lateral and ventral midline domains, with the major contribution to the anterior bud coming from the ventral midline precursors (Angelo et al., 2012; Tremblay and Zaret, 2005). Thus, the domains are differentially exposed to BMP and FGF-MAPK signaling prior to merging in a single domain of homogeneously active FGF signaling.

BMP signaling from septum transversum mesenchyme and lateral plate mesoderm also has hepatic induction capacity (Rossi et al., 2001; Shin et al., 2007; Zhang et al., 2004). Lineage-tracing studies in zebrafish revealed a hepato-pancreatic progenitor, and it was shown that Bmp2 signaling downregulates the expression of *pdx1*, which encodes a transcription factor involved in pancreatic development, in this progenitor, but is not required to maintain the hepatic program (Chung et al., 2008). BMP2a, on the other hand, is required for continued proper expression of the liver program (Naye et al., 2012). How BMP signaling interacts with the FGF and Wnt pathways is unclear, but FGF signaling does not function downstream of BMP, and Wnt signaling does not lie downstream of BMP or FGF signaling.

Finally, Wnt/β-catenin signaling is also essential for hepatic specification (McLin et al., 2007; Negishi et al., 2010; Ober et al., 2006). In zebrafish, Wnt2bb is expressed in the anterior lateral plate mesoderm and is necessary for hepatoblast gene expression; loss of Wnt2 and Wnt2bb results in liver agenesis (Poulain and Ober, 2011). Conversely, constitutively active β-catenin results in ectopic liver formation in zebrafish by conversion of pancreatic progenitors to hepatoblasts prior to liver specification (So et al., 2013). In contrast to BMP or FGF signaling, Wnt/β-catenin signaling can even induce conversion of non-hepatoblast cells, such as intestinal progenitors, into hepatoblasts in zebrafish. Surprisingly, studies have not revealed a clear function for Wnt during hepatic specification in mice. Although *Wnt2* and *Wnt2b* are expressed in the ventral mesoderm surrounding the anterior foregut from E9.0 to E10.5, *Wnt2* loss only causes lung hypoplasia, and *Wnt2b*-null embryonic livers are normal (Goss et al., 2009; Tsukiyama and Yamaguchi, 2012). Furthermore, the combined loss of *Wnt2* and *Wnt2b* causes lung agenesis without affecting other endoderm-derived organs (Goss et al., 2009). Nonetheless, co-culture experiments using endoderm cells differentiated from mouse ESCs or harvested from E8.25 embryos showed that the canonical Wnt pathway together with the Notch pathway must be repressed to allow endothelial cell-dependent hepatic specification (Han et al., 2011). In addition, non-canonical Wnt signaling components are expressed in foregut and pancreas progenitors but are strongly downregulated in liver progenitors (Rodriguez-Seguel et al., 2013). Concordantly, molecules associated with non-canonical Wnt signaling, such as FZD2, ROR2, CELSR2 and FAT1, are enriched in foregut epithelial and pancreatic progenitors but absent from hepatic progenitors at E8.5 and E10.5. In addition, *Wnt5a* is expressed at E8.5 in the foregut; later, it is maintained in pancreatic progenitors but absent in hepatoblasts. Activation of the non-canonical Wnt pathway using recombinant WNT5a and WNT5b proteins or RNA injection in *Xenopus* embryos induced a strong pancreatic progenitor program, including in the hepatic region where there was a downregulation of early liver markers (Rodriguez-Seguel et al., 2013)*.*

Thus, key questions remain unanswered regarding the role of Wnt signaling during liver specification. In the future it will be important to determine whether the Wnt pathway plays a conserved role in mammals, and to achieve a deeper understanding of the interplay between the canonical and non-canonical pathway branches at this stage of liver development.

### Signaling during hepatoblast expansion and differentiation

Hepatoblast expansion is regulated by interactions with surrounding endothelial and mesenchymal cells in the septum transversum (Shin and Monga, 2013), and again Wnt signaling is a key positive regulator. In mice, nuclear β-catenin levels peak at E10-E12, correlating with the number of proliferating cells (Micsenyi et al., 2004). The expression of constitutively active β-catenin leads to liver enlargement at E15, whereas inhibition results in a small liver due to decreased cell proliferation and increased apoptosis (Suksaweang et al., 2004). It was also shown that Wnt signaling promotes the proliferation of hepatoblasts (Goessling et al., 2008; McLin et al., 2007), acting in collaboration with hepatocyte growth factor (HGF) and FGF signaling (Berg et al., 2007; Schmidt et al., 1995). Wnt signaling can be activated by the phosphorylation of β-catenin by the HGF receptor MET or by nuclear translocation caused by FGF signaling.

Bipotential hepatoblasts differentiate to hepatocytes and biliary cells in the vicinity of the portal mesenchyme, and this process is regulated by TGFβ, Notch, Wnt, BMP and FGF. A porto-parenchymal gradient of TGFβ activity is generated by the expression of TGFβ2 and TGFβ3 in the periportal region, resulting in a peak of TGFβ signaling activity around the portal mesenchyme (Antoniou et al., 2009; Clotman et al., 2005). A role for this pathway in the maturation of the developing ducts at later stages has also been proposed (Lemaigre, 2010). Several studies have linked Notch signaling to hepatocyte differentiation. For example, mutations in *NOTCH2* or *JAG1*, which encodes a Notch ligand, are associated with the paucity of bile ducts in Alagille syndrome (Li et al., 1997; McDaniell et al., 2006). It was also shown that the inhibition of Notch signaling results in reduced biliary differentiation, whereas activation promotes biliary differentiation from hepatoblasts (Tchorz et al., 2009; Zong et al., 2009). Hepatoblasts also require the Notch mediator RBPJ and the Notch target gene *Hes1*, and biliary tubulogenesis requires Notch signaling (Lemaigre, 2010). However, when Notch signaling is impaired before hepatoblast differentiation, a more severe postnatal phenotype is observed (Falix et al., 2014; Jeliazkova et al., 2013), suggesting that cholangiocyte differentiation becomes less dependent on *Notch2* as development proceeds.

Wnt signaling also promotes cholangiocyte specification, proliferation and survival. The stabilization of β-catenin promotes biliary differentiation and its deletion results in defects in bile duct formation and increased apoptosis of hepatoblasts in mid-gestational mouse fetal livers (Decaens et al., 2008; Tan et al., 2008). Two isoforms of β-catenin are found during early liver development (Lade et al., 2012); only the predominant full-length form is found in biliary epithelial cells, whereas a truncated β-catenin localizes to the membranes and nuclei of hepatocytes. The non-canonical Wnt pathway is also implicated in biliary development, as disruption in the Wnt/planar cell polarity (PCP) pathway impairs bile duct formation (Cui et al., 2011). Furthermore, the knockout of non-canonical *Wnt5a* causes increased numbers of biliary precursor cells and promotes the expression of biliary markers (Kiyohashi et al., 2013). In this context, WNT5a signaling increases the phosphorylation of calcium/calmodulin-dependent protein kinase II (CaMKII) downstream of non-canonical Wnt signaling in the fetal liver, which suppresses biliary differentiation.

### Hepatic artery-bile duct cross-talk

During hepatogenesis, the developing bile ducts remain closely associated with hepatic arteries, and signaling between these two tissues regulates their coordinated development. For example, VEGF generated from cholangiocytes and angiopoietin 1 derived from hepatoblasts work together to support angiogenesis and remodel the portal vasculature in order to support the developing epithelium (Fabris et al., 2008). The timing depends on species, as bile duct development in mice precedes hepatic artery development, whereas it is the opposite in humans. Furthermore, the zonal characteristics of the hepatic lobule are influenced by the architecture of the hepatic vascular system, and this co-patterning is dependent on Wnt/β-catenin, Notch and VEGF signaling (reviewed by Cast et al., 2014).

### Signaling during hepatocyte maturation and liver growth

Factors promoting hepatocyte maturation include OSM, glucocorticoids, HGF and Wnt, whereas TNFα antagonizes OSM (Shin and Monga, 2013). Also implicated is the Hippo/Yap signaling pathway, a crucial regulator of liver size (Camargo et al., 2007; Dong et al., 2007). Within this pathway, the tumor suppressor NF2 (merlin) and the proto-oncogene YAP1 are antagonistic with respect to liver growth (Zhang et al., 2010); loss of YAP1 in the liver decreases hepatocyte survival and causes a profound defect in biliary cell development, whereas loss of NF2 causes hyperplasia of both cell types. In the mature liver, conditional activation of nuclear YAP1 induces hepatocyte dedifferentiation to a progenitor ‘oval’ cell phenotype, and this transition is mediated via Notch signaling (Yimlamai et al., 2014).

## The genetic and epigenetic control of liver development

A number of transcription factors and epigenetic regulators have been implicated in liver development. Notably, at the hierarchical apex for specification of prehepatic fate are sister genes from the FoxA and GATA families of nuclear DNA-binding proteins.

### FOXA1/2/3

DNA binding activities that were initially discovered in hepatocyte cell lysates were collectively assigned to proteins termed hepatic nuclear factors (HNFs). These in fact belong to various transcription factor gene families including the FoxA family (HNF3), the Cut-homeodomain family (HNF6), the bZIP family (CEBPα), the Pou-homeodomain family (HNF1α) and orphan receptors (HNF4α). The FoxA family members FOXA1/2/3 possess DNA-binding domains containing a ‘forkhead box’ (FOX) helix-loop-helix that includes extended loop or ‘winged helix’ structures, similar to those of linker histones (Kaestner, 2010). This structural aspect imparts the capacity to interact directly with compacted nucleosomes in chromatin, a feature that led to the designation of FoxA proteins (along with GATA4, see below) as ‘pioneer factors’, which are able to seed the transcriptional and epigenetic complexes of liver-specific genes (Cirillo et al., 2002).

*Foxa1* is expressed in prehepatic foregut endoderm but is not required for liver development. *Foxa2* is expressed earlier, at ∼E6.5 in the primitive streak and node, and *Foxa2* knockout embryos die by E9, lacking foregut endoderm and notochord (Ang and Rossant, 1994; Weinstein et al., 1994). FOXA3 is also expressed in foregut endoderm (Monaghan et al., 1993), but *Foxa3*-null mice can be maintained. Thus, the *Foxa3* promoter has been used as an ideal Cre-driver for the conditional depletion of other foregut genes. When this approach was used to delete *Foxa2* in foregut endoderm, the loss of *Foxa2* was shown to be compensated by *Foxa1*. Indeed, hepatic specification fails when *Foxa2* is deleted conditionally in foregut endoderm in a *Foxa1* mutant background (Lee et al., 2005). The resulting mutant endoderm lacks competence for hepatic fate, even if treated *in vitro* with FGF. It was also shown that *Foxa1/2* activity in the anterior foregut endoderm is negatively regulated by Groucho co-repressors, and that forced expression of GRG3 (TLE1) is sufficient to block hepatic differentiation in embryonic foregut explants (Santisteban et al., 2010), suggesting that FoxA-dependent chromatin domains are established but non-functional until GRG3 co-repressor levels are reduced and replaced by productive activators. In line with this, through a mechanism termed ‘bookmarking’, FOXA1 remains associated non-specifically with chromatin during mitosis, as well as remaining bound at a subset of specific cognate sites important for regulating liver differentiation (Caravaca et al., 2013). This bookmarking facilitates efficient reactivation of the hepatocyte program as cells complete the mitotic cycle.

### GATA4/5/6

Initial studies revealed important functions for GATA4 in cardiogenesis, although GATA4 and its close relatives GATA5 and GATA6 are also expressed and play important roles in gut organ derivatives including the liver (Laverriere et al., 1994). GATA factors therefore regulate the simultaneous development of two closely associated organs (heart and liver) that are derived from distinct germ layers (mesoderm and endoderm, respectively). Like FOXA1, GATA4 has the inherent capacity to interact with compact chromatin and is bound to the *Alb* enhancer in prehepatic endoderm as a pioneer factor (Cirillo et al., 2002). Also like FoxA factors, GATA factors are co-expressed in the foregut endoderm and display both redundant as well as gene-specific functions during gut organogenesis. In zebrafish, the depletion of *gata4*, *gata5* or *gata6* causes a severe defect in expansion of the liver bud, although the bud is specified normally (Holtzinger and Evans, 2005; Reiter et al., 2001). Furthermore, the co-depletion of any two factors blocks hepatic specification (Holtzinger and Evans, 2005), demonstrating functional redundancy. Extensive gene profiling studies implicate Gata4/5/6 in a ‘core’ DE program comprising multiple autoregulatory and cross-regulatory loops (Sinner et al., 2006).

Analyses of GATA functions in the mouse liver have been complicated by earlier essential roles in extra-embryonic endoderm (ExEn) development. However, using tetraploid embryo complementation to rescue the ExEn defect, it has been shown that *Gata4* and *Gata6* do function in liver development (Watt et al., 2007; Zhao et al., 2005). As in fish, the loss of either gene in the mouse embryo proper impairs liver bud expansion, although hepatic endoderm is specified. It is expected that loss of both genes would eliminate hepatic specification, although this has not been formally tested. *Gata5* does not appear to function in mouse liver development, but the same was thought true for GATA5 in heart development until mutant *Gata5* alleles were combined with mutant alleles for *Gata4* or *Gata6* (Singh et al., 2010). Overall, the data suggest early redundant functions for *Gata4/5/6* during the specification of hepatic endoderm (and perhaps foregut endoderm) and subsequent non-redundant roles in liver bud growth. Whether these factors regulate the same or different genes remains an open question.

In purified adult hepatocytes GATA4 is bound at a large number of genes that control key aspects of liver physiology (Zheng et al., 2013). Nevertheless, the loss of *Gata4* or even of *Gata4* and *Gata6* in differentiated hepatocytes causes surprisingly few changes in gene expression or liver function. This suggests that, once an active transcriptional complex is formed, other factors such as FOXA1 might be sufficient to bookmark the regulatory elements and maintain the hepatic program. However, it should be noted that a potential compensatory role for *Gata5*, which is important for liver development in zebrafish, has not been ruled out.

### Homeobox genes

*Hhex* encodes a homeodomain protein that is expressed in both hepatic cells and biliary epithelium and is required for liver bud morphogenesis. In the *Hhex* mutant embryo, the hepatic diverticulum is specified but cells fail to proliferate normally or move into the mesenchyme (Bort et al., 2006). The conditional depletion of *Hhex* in the hepatic diverticulum (achieved using *Foxa3:cre*) demonstrated its requirement for the normal expression of *Hnf4a*, *Hnf6* (*Onecut1*) and *Hnf1b*, and mutants develop hypoplastic cystic livers (Hunter et al., 2007). Mice deleted for *Hhex* in hepatoblasts (*Afp:cre*) survive but fail to maintain *Hnf1b* expression and exhibit hyperproliferative biliary epithelial cells. Like GATA4/6, the homeodomain protein HNF1β is required for ExEn development, but a subsequent function for it in hepatic specification was demonstrated using tetraploid embryo complementation of the null allele (Lokmane et al., 2008). These *Hnf1b* mutant embryos develop hypoplastic liver lobes lacking hepatoblasts and fail to express ALB. HNF1β appears to be downstream of *Gata6* and is required for thickening of the hepatic bud and to maintain the expression of *Foxa1/2/3*. The subsequent delamination and migration of the hepatic progenitors into stroma requires at least one of two functionally redundant homeodomain factors HNF6 and ONECUT2 (Margagliotti et al., 2007), as well as the prospero homeoprotein PROX1 (Sosa-Pineda et al., 2000).

### Transcription factor function and cooperation during hepatoblast differentiation

A chromatin immunoprecipitation (ChIP)-based study evaluating the recruitment of factors to hepatic transcription factor promoters has shown that the differentiation of specified hepatoblasts is regulated by a core group of transcription factors (Kyrmizi et al., 2006) that includes FOXA2, HNF1β, HNF1α, HNF4α, HNF6 and NR5A2. In addition, embryos lacking *Hnf4a* fail to express markers of differentiated hepatocytes in the liver bud, while liver architecture (including the organization of endothelial cells) is disrupted in hepatoblasts lacking *Hnf4a* (Parviz et al., 2003). During hESC-directed differentiation, blocking HNF4α expression with shRNA blocks the earlier step of hepatic specification as well as the expression of *FOXA1/2* and *GATA4/6* (DeLaForest et al., 2011). This might represent a difference between the human and mouse systems or a more rigorous requirement for HNF4α in the setting of *in vitro* differentiation. The homeobox gene *Prox1* also modulates hepatoblast differentiation; when it is deleted from bipotent hepatoblasts, ectopic bile ducts develop at the expense of liver parenchyma, while in periportal areas the progenitors generate excessive cholangiocytes and biliary hyperplasia (Seth et al., 2014).

*Foxa2* also appears to act in parallel with *Sox17* in controlling foregut and mid-hindgut endoderm formation (Viotti et al., 2014).*Sox17* expression is associated with the specification of DE (Sinner et al., 2006). Genetic studies suggest a primary function during specification of the extrahepatobiliary system from PDX1^+^ pancreatobiliary bipotential progenitors, and *Sox17* is downregulated in the field of ventral foregut endoderm as hepatic fate is specified (Spence et al., 2009). Liver differentiation genes, including *Hhex*, *Hnf6* and *Hnf4a*, are also implicated in biliary tree development. Thus, overlapping patterns (and perhaps levels) of the same recurring set of transcription factors control the fate of closely related hepatic, pancreatic and biliary lineages.

### Stability and flexibility of the liver transcription factor network

FoxA and GATA family members are also essential for specification of the ventral pancreas. Hence, foregut endoderm marked by *Hhex* expression is first subdivided by homeobox genes into hepatic (HNF1β-expressing) or pancreatic (PDX1-expressing) domains. Bipotential progenitors in the endoderm are ‘poised’ to commit to either fate based on differentially placed epigenetic marks that are present on hepatic or pancreatic gene regulatory elements (Xu et al., 2011). For example, acetylated H3K9K14, which is typically associated with active genes, is enriched at the upstream regulatory elements of the silent *Pdx1* gene as compared with silent liver gene regulatory regions. Yet the trimethylated H3K27 mark associated with repression and the EZH2 methyltransferase are also preferentially associated with *Pdx1*. Thus, *Pdx1* is ‘pre-patterned’ to be either activated or repressed as an endoderm progenitor commits to a hepatic or pancreatic fate. Anterior ventral foregut endoderm appears to be primed by default toward pancreatic fate, with p300 (Ep300)-dependent histone acetylation required to activate the hepatic program (Xu et al., 2011). *Ezh2* continues to function in committed embryonic hepatic cells for their growth and maturation and, accordingly, conditional knockout of *Ezh2* at E8.5 causes a substantial loss of liver tissue, whereas knockout from E13.5 partially inhibits hepatocyte differentiation (Koike et al., 2014).

*Hhex*^+^ progenitors require *Hnf6* and *Prox1* for their migration, but these genes are then differentially required for specifying hepatic (*Prox1*, *Hnf4a*) or cholangiocyte (*Hnf6*, *Hnf1b*, *Sox9*) fate. This switch is partially controlled by *Tbx3*, which supports *Prox1* expression and the repression of *Hfn6*/*Hnf1b* in the liver bud (Lüdtke et al., 2009). In line with this, loss of *Prox1* in the hepatoblast causes an expansion of biliary cells at the expense of hepatocytes (Seth et al., 2014), whereas *Hnf1b* knockout causes ductopenia and bile duct dysplasia (Coffinier et al., 2002). This appears to reflect progenitor output, rather than reprogramming, since depletion of *Prox1* in committed hepatocytes does not cause biliary hyperplasia. In addition, patients heterozygous for *HNF1B* (suffering from renal cyst and diabetes syndrome) have normal hepatocyte function but ciliary defects associated with cholestasis (Roelandt et al., 2012).

MicroRNAs have also been implicated during liver development. HNF6 and probably other hepatic transcription factors regulate the expression of miR122, which acts in a positive-feedback loop to induce hepatocyte differentiation (Laudadio et al., 2012). The mechanism may involve downmodulation of the repressor protein CUTL1 (CUX1) (Xu et al., 2010), and grainyhead-like 2 balances this loop by repressing the expression levels of miR122 in neonatal bipotential hepatoblasts (Tanimizu et al., 2014).

## Linking signaling to transcriptional regulators

Developmental signaling is required to activate the expression of key transcription factors, but these factors then feedback into the signaling programs to coordinate developmental transitions and stabilize gene regulatory networks. During endoderm patterning, high Nodal activity correlates with the expression and function of *Foxa2* and *Sox17*. Wnt/β-catenin is also necessary for *Sox17* expression and function in the DE (Engert et al., 2013; Sinner et al., 2004). Explant studies in mice have shown that BMP from the septum transversum mesenchyme activates the expression of *Gata4* (Rossi et al., 2001) and is associated with the specification of hepatic endoderm. In addition, GATA6 may maintain a regulatory loop as an upstream activator of BMPs (Peterkin et al., 2003; Rong et al., 2012). The cardiogenic mesoderm may also provide (in addition to BMP) an FGF signal that is important for development of the anterior liver bud (Wang et al., 2015). During gastrulation, FGF4 in the endoderm promotes a more posterior gut fate by activating *Pdx1* and *CdxB* expression while repressing the more anterior foregut fates (Dessimoz et al., 2006). However, using heat shock-inducible transgenes to block BMP or FGF signals, both pathways were shown to be essential for *hhex* and *prox1* expression and to promote hepatic specification in zebrafish (Shin et al., 2007).

A number of studies suggest that the extracellular matrix (ECM) facilitates BMP, Wnt and FGF cross-regulation. For example, BMP in the *Xenopus* foregut is modulated by an autoregulatory loop that induces Sizzled, a secreted Frizzled-related protein (SFRP) that inhibits BMP signaling, which in turn negatively regulates Tolloid metalloproteases and controls fibronectin (FN) deposition between the endoderm and mesoderm (Kenny et al., 2012). *Xenopus* Sizzled morphants exhibit reduced Wnt signaling in the foregut, while Sfrp5 in the anterior region of zebrafish modulates Wnt and BMP signaling via Tolloid inhibition (Kenny et al., 2012; Stuckenholz et al., 2013). It has also been suggested from ESC-based modeling that an FGF-dependent FN gradient exists in the early mouse embryo, with lower FN levels in the anterior endoderm (Villegas et al., 2013). *Fn1*-null embryos show an expanded *Cer1* anterior region, suggesting that *Fn1* helps restrict the prospective foregut domain. *Sox17* expression is also inversely correlated with *Cer1* expression, although *Fn1* expression is normal in *Sox17* mutants, in which the basement membrane between mesoderm and endoderm is absent and the posterior foregut is impaired, raising the possibility that other components of the ECM are involved (Viotti et al., 2014). The timing of active signaling relative to the expression of key transcription factors was mapped by analyzing downstream signals, for example phosphorylated ERK1/2 (MAPK3/1) for FGF and phosphorylated SMAD1/5/8 for BMPs (Wandzioch and Zaret, 2009). This study showed that BMP initially activates a hepatic (*Prox1*) program while repressing pancreatic (*Pdx1*) fate. However, only a few hours later, by the 5- to 6-somite stage, BMP activates both programs while FGF represses *Pdx1*. Active TGFβ signaling is generally repressive of both programs, suppressing the expression of *Hnf1b*.

The Hippo pathway has also been linked to the control of the liver transcriptional network. By mapping shared HNF4α and FOXA2 binding regions, which are predicted to represent enhancers, Hippo signaling was implicated in the transition from hepatoblast to differentiated hepatocyte (Alder et al., 2014). Over half the sites changed in the differentiated cells via ‘enhancer switching’. Only the regions bound in hepatoblasts were also enriched for a motif that binds the transcription factor TEAD2, which functions with its coactivator YAP1. Sphingosine 1-phosphate signaling, acting via LATS1/2 and YAP1, is also essential for proper anterior endoderm formation and might cell-autonomously regulate endodermal cell attachment to the ECM by regulating the transcription of connective tissue growth factor (CTGF), which is known to interact with FN1 (Fukui et al., 2014).

## Future perspectives

Although the liver is a naturally regenerative organ, transplantation currently remains the only curative therapy when the liver is damaged to the point of failure. There is thus great interest in translating our understanding of liver development into regenerative therapies, and this knowledge has already facilitated efficient protocols for generating hepatic cells from human pluripotent stem cells *in vitro*. In addition, hepatic transcription factors that regulate liver development have been used to directly reprogram human fibroblasts to hepatocytes (Du et al., 2014; Huang et al., 2014), although this is relatively inefficient and cells only expand by expressing oncogenes. The concept of hepatocyte transplantation also faces many challenges and, thus far, has shown limited efficacy (Fox et al., 2014). It might therefore be useful to focus on two additional areas of research. First, the generation and rigorously defined phenotyping of earlier progenitors, especially if these can be expanded (without oncogenes) and can provide supportive tissue and niche components, including vascular and biliary cells. Second, given the advances in understanding the developmental programs that generate the liver, it is now reasonable to apply this knowledge to tissue engineering strategies for creating functional liver as replacement tissue.

Using directed differentiation from pluripotent cell sources, hepatic cells can now be grown in culture at scales that could be therapeutic. Yet, given the complexity of liver architecture, it is perhaps not surprising that cellular transplantation to treat human liver failure has not been successful. Efforts have been made to generate extra-corporeal bioartificial liver (BAL) devices that increase hepatocyte survival and blood detoxification, although these have had relatively limited clinical success. The capacity for a stem/progenitor cell to impact adult liver function has also been controversial and might be limited (Miyajima et al., 2014). An alternative approach is to enhance the innate regenerative capacity of the liver, and a better understanding of hepatocyte/cholangiocyte maturation might provide clues for releasing the postmitotic proliferative potential of liver cells, even in damaged tissue. This is likely to involve the same developmental signaling pathways that function during embryonic hepatogenesis, as demonstrated by the requirement of Wnt/β-catenin for regeneration in animal models (Boulter et al., 2012; Tan et al., 2006). With improved understanding of progenitor relationships and fates, particularly in humans, and with the development of humanized mouse models, it might be possible to better organize small liver organoids that have enough supportive environment to survive and that can be scaled or grown in culture for use in either BAL or transplant therapies. Although the cirrhotic liver is not an environment that tolerates transplanted cells/tissues, other internal organs might provide sufficient vascular support, as shown, for example, by the successful transplantation of functional hepatocytes into murine lymph nodes (Komori et al., 2012) or of self-assembled organoids into the mesentery (Takebe et al., 2013).

Tissue engineering strategies might also help to overcome the challenge of strict dependency on the microenvironment for hepatic function, as recently reviewed (Bhatia, 2014). However, a major gap in our knowledge is how to specify the zonal phenotypes that are imparted by hepatic lobule architecture. This is surely impacted by the intricate structure of the lobules, with their arranged cords of polarized hepatocytes that interact with ECM and other cell types. Understanding this problem will benefit from progress in identifying specific signaling components (ligands, receptors, transcription factors) that mediate the cell fate transitions during liver development. However, it is possible that co-culture systems, tissue printing processes or 3D bio-scaffolds might be needed to replicate some aspects of tissue architecture, and this will be a challenge to scale. Animal models that better replicate the clinical manifestations of acute liver failure (such as intracranial hypertension) also need to be developed in order to test and optimize such strategies. Together, these multidisciplinary approaches will hopefully provide a more complete understanding of the orchestration of liver development, both *in vivo* and *in vitro*.
